# Influence of Crosslink Density and Stiffness on Mechanical Properties of Type I Collagen Gel

**DOI:** 10.3390/ma8020551

**Published:** 2015-02-06

**Authors:** Shengmao Lin, Linxia Gu

**Affiliations:** 1 Department of Mechanical and Materials Engineering, University of Nebraska-Lincoln, Lincoln, NE 68588-0656, USA; E-Mail: linshengmao@gmail.com; 2 Nebraska Center for Materials and Nanoscience, Lincoln, NE 68588-0656, USA

**Keywords:** crosslink density, stiffness, collagen gel, polymerization, tissue engineering

## Abstract

The mechanical properties of type I collagen gel vary due to different polymerization parameters. In this work, the role of crosslinks in terms of density and stiffness on the macroscopic behavior of collagen gel were investigated through computational modeling. The collagen fiber network was developed in a representative volume element, which used the inter-fiber spacing to regulate the crosslink density. The obtained tensile behavior of collagen gel was validated against published experimental data. Results suggest that the cross-linked fiber alignment dominated the strain stiffening effect of the collagen gel. In addition, the gel stiffness was enhanced approximately 40 times as the crosslink density doubled. The non-affine deformation was reduced with the increased crosslink density. A positive bilinear correlation between the crosslink density and gel stiffness was obtained. On the other hand, the crosslink stiffness had much less impact on the gel stiffness. This work could enhance our understanding of collagen gel mechanics and shed lights on designing future clinical relevant biomaterials with better control of polymerization parameters.

## 1. Introduction

Type I collagen network, a major component of the extracellular matrix (ECM) of connective tissues, has a profound impact on cellular and tissue behaviors. Type I collagen gels are widely used as a three-dimensional (3D) scaffold for culturing cells and engineering various tissues capable of providing optimal microenvironments in the form of physical and chemical cues [1]. The structural properties of collagen gel provide the basis of cell-scaffold interactions and were considered in many scaffold designs [2]. 

It was well acknowledged that microstructure configurations modulated the macroscopic properties of cross-linked fiber networks [3]. The relationship between mechanical properties of collagen gel and the quality of cross-linked fiber structure, (including fiber dimensions, fiber strength, and various polymerization reaction conditions including collagen concentration, pH, *etc*.) was documented in the literature [2,4]. Experimental studies [5,6,7] have shown that type I collagen gel stiffness and failure stress increased with collagen concentration, pH, or temperature during polymerization. These polymerization conditions also led to an increased fiber density, fiber length or a reduced cross-section. In addition, Zeugolis *et al*. showed that the chemical crosslinking potently altered the gel stiffness and failure stress more than physical or biological crosslinking approaches [8]. Sheu *et al*. observed that the concentration of glutaraldehyde was positively correlated with the degree of cross-linking, (*i.e.*, crosslink density) [9]. Charulatha *et al*. demonstrated that five cross-linking agents led to different crosslink density and chemical structure, as well as mechanical responses of formulated collagen membrane [10]. The various chemical structures of crosslinks are speculated to correspond to different tensile strength. 

Computational models of random distributed fibers were also utilized to further inspect the mechanism of fiber network behaviors for fine-tuning the microenvironment of cell culture [11,12]. Crosslinks in two-dimensional models was simply represented as intersection points, which were constrained either as freely rotating pin joints [13,14,15] or welded joints [16]. The 3D crosslinks were treated as either regular fibers [12] or torsional springs where their rotational stiffness was obtained by fitting to experimental data [11]. However, the role of crosslinks on the gel mechanics was not elucidated yet in the existing models. 

In this work, the role of crosslinks on the collagen gel properties was investigated through computational modeling. The collagen fiber network modulated gel behavior was validated against the experiment by Roeder *et al*. [6]. The mechanism of strain stiffening of collagen gel was elucidated. The crosslinks with varied density and stiffness corresponding to different polymerization conditions [9,10] were formulated in a 3D collagen network. These microscopic crosslink properties were then correlated with the macroscopic gel mechanics. These results could be used to guide the design of scaffold with tunable material properties. 

## 2. Materials and Methods

**Figure 1:**
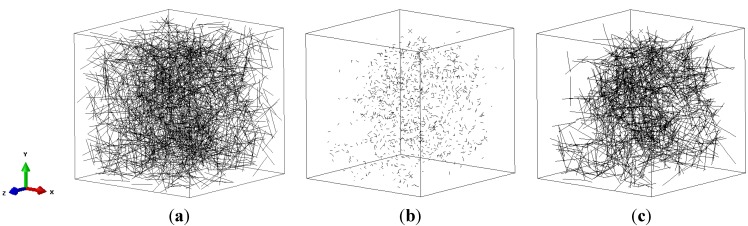
Representative volume element (RVE) with random distributed fibers (**a**) Before cross-linking; (**b**) Crosslinks; (**c**) Cross-linked fiber network.

A representative volume element (RVE) with the side length of 40 μm was used to represent commonly used type I collagen gel with a concentration of 1 mg/mL [17], which is equivalent to the fiber volume fraction of 0.073% (Figure 1a). 3D collagen fibers (1934 in total) were randomly distributed using the random seed algorithm [18]. The fiber is 8 μm long and could be truncated to 4 μm at the boundaries. The fiber diameter of 62 nm was based on the measurement for the collagen gel polymerized at 37 °C and pH 7.4 [5]. Each fiber was meshed with 2 μm beam elements corresponding to the mean crosslink spacing of collagen fiber networks [19]. The crosslinks were generated between nodes when their distance is less than or equal to a certain value, referred to as crosslink threshold. Figure 1b demonstrated 2360 crosslinks between fibers in Figure 1a with a threshold of 800 nm. The uncross-linked fibers were then removed, shown in Figure 1c, due to lack of contribution to the mechanics of collagen networking. The crosslink density was calculated as the number of crosslinks per collagen fiber, (e.g., 2.09 in Figure 1c). 

The Young’s modulus of collagen fiber was adopted as 50 MPa [11]. Crosslinks were assumed to have the same material property as collagen fibers in the baseline model. Uniaxial tension was applied along the x-direction of the RVE. No sliding motion existed between crosslink and collagen. Nonlinear finite element models were solved using ABAQUS 6.12 (Simulia, Providence, RI, USA). Various crosslink thresholds and crosslink stiffness were tested to unravel the role of crosslink on type I collagen gel properties, (*i.e.*, the fiber network properties). 

The orientation of collagen fiber network was analyzed using the OrientationJ plugin [20] in ImageJ software (NIH, Bethesda, MD, USA). The non-affine deformation of fiber network is quantified as *S* [21]:

(1) $$S=\sqrt{\frac{1}{N}\sum_{i=1}^{N}{\left(\frac{d_{i}-x_{i}\varepsilon }{x_{i}\varepsilon }\right)}^{2}}$$

where *d_i_* is the displacement for the *i*th node located at *x_i_* from previous step when the network has a macroscopic strain value of ε. A larger *S* indicates an increased non-affinie deformation of fiber networks, with 0 as an affine deformation. 

## 3. Results

### 3.1. Model Validation

**Figure 2:**
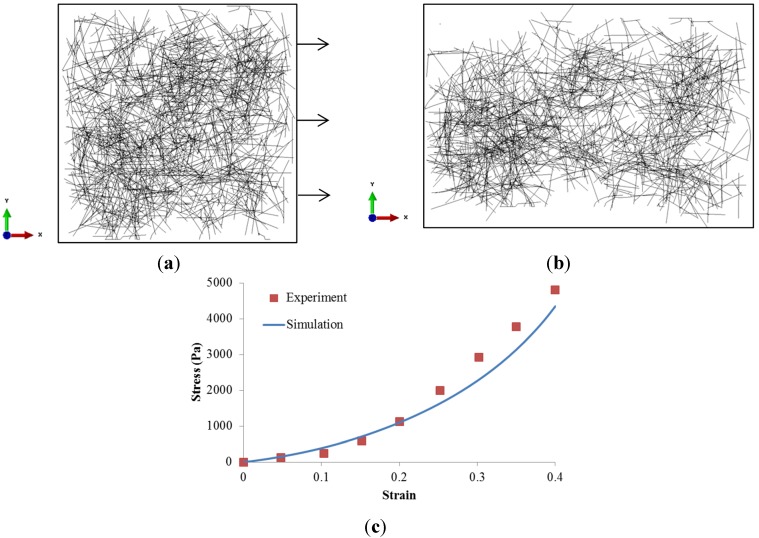
Cross-linked collagen fiber network (**a**) at zero loading; (**b**) at 40% strain along *x*-direction; and (**c**) stress–strain relationship.

The experimental work by Roeder *et al*. [6] was simulated using our RVE model subjected to 40% strain along *x*-direction as shown in Figure 2a. The fiber diameter was measured as 435 nm with the Young’s modulus of 79 MPa, and the crosslink threshold was assumed as 450 nm. The fiber network strain was estimated from the relative edge displacement. The fiber network stress along *x*-direction was calculated by the edge reaction force divided by the total fiber cross-section area on the *y*–*z* plane. The stress-strain relationship of 3D collagen gel was depicted in Figure 2c, with comparison to the experimental measurements. It was clear that our RVE simulation agreed well with the experiments, especially at strain less than 20%. The discrepancy at larger strain could be explained by the actual heterogeneous fiber dimensions.

### 3.2. Strain Stiffening Effect in the Baseline Model

**Figure 3:**
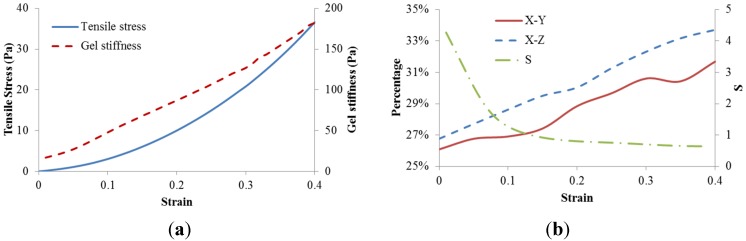
Dynamic results of baseline model. (**a**) Stress–strain relationship and gel stiffness along loading direction; (**b**) Percentage distribution of fibers in both *x*–*y* and *x*–*z* planes within −15 to +15 degree angle relative to the loading axis, as well as the non-affine parameter *S*.

Even though both collagen fibers and crosslinks were modeled as linear elastic materials, the fiber network exhibited obvious strain stiffening (Figure 3a). It was clear that the network stiffness was increased with strain, and its magnitude is much less than the stiffness of either fibers and crosslinks due to low fiber volume fraction. This could be explained by the fiber alignment. Therefore the orientation of collagen fibers as well as the non-affine motion property *S* was monitored. The collagen fiber orientation was quantified as the percentage distribution of collagen fibers within −15 to +15 degree angle relative to the loading axis *x*, which shows the occurrences of fiber alignment along the loading axis at a defined range as a precentage of the total number of fibers. It was shown in Figure 3b that fiber alignment along loading axis in both *xy* and *xz* planes increased with a larger strain. It was also found that the non-affine deformation parameter *S* decreased with the increased strain. The non-affine deformation was dominated at strains less than 10% which correspond to the reorganization of random distributed collagen fibers. As strain exceeded 10%, the fiber alignment along the loading axis continued to increase with the strain, however, the network deformation tended to be more affine. 

### 3.3. Effect of Crosslink Density 

**Figure 4:**
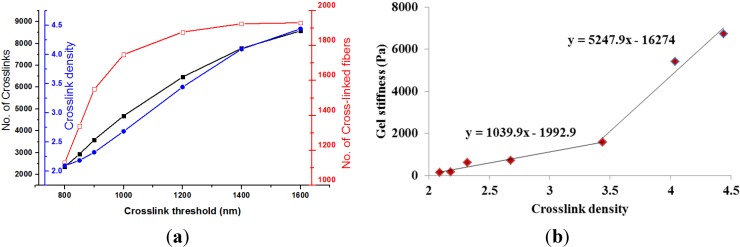
(**a**) Crosslink threshold regulated microstructure changes of collagen gel; (**b**) Correlation between crosslink density and gel stiffness.

In this work, crosslink density was regulated by the crosslink threshold as listed in seven RVE models in Table 1. As the crosslink threshold, (*i.e.*, maximum crosslink distance), increased from 800 nm in the baseline model to 1600 nm, the number of crosslinks surged from 2360 to 8572, however the number of cross-linked fibers only increased from 1130 to 1931, leading to crosslink density varying from 2.09 to 4.439. The crosslink threshold regulated microscale fiber network configurations was also depicted in Figure 4a. The increase of crosslink threshold resulted in larger numbers of crosslinks and crosslink density as well. However, a plateau was clearly observed for the number of cross-linked fibers as the maximum crosslink distance exceeded 1200 nm. This indicated a fully cross-linked collagen fiber network. Figure 4b plotted the relationship between the crosslink density and collagen gel stiffness. It was clearly observed that the gel stiffness increased with crosslink density. Specifically, a bilinear relationship was obtained. The rate of stiffness growth increased almost four times when the crosslink density was larger than 3.44, corresponding to the crosslink threshold of 1200 nm. 

**Table 1:** RVE models with different crosslink densities.

Cases	Base	1	2	3	4	5	6
Crosslink threshold (nm)	800	850	900	1000	1200	1400	1600
No. of Crosslinks	2360	2933	3594	4687	6467	7776	8572
No. of Cross-linked fibers	1130	1340	1550	1749	1878	1925	1931
Crosslink density	2.09	2.18	2.32	2.68	3.44	4.039	4.439
Gel stiffness (Pa)	30.02	40.823	154.27	545.3	1280.3	4379.6	5659.1

### 3.4. Effect of Crosslink Stiffness 

Crosslink stiffness was varied from 50 MPa in the baseline model to 25 MPa, 75 MPa and 100 MPa for studying its role on the type I collagen gel behavior. It was clear from Table 2 that stiffer crosslinks resulted in increased gel stiffness. This could be attributed to the increased load sharing capacity of crosslinks. It was shown from our models that the percentage of load shared by crosslinks was increased with the crosslink stiffness; however its share on the percentage of strain energy was reduced. 

**Table 2:** Role of crosslink stiffness on Load sharing capacity of crosslinks.

Crosslink Stiffness	25 MPa	50 MPa	75 MPa	100 MPa
Gel stiffness	23.3 Pa	30.0 Pa	31.4 Pa	32.2 Pa
Percentage of total load shared by crosslinks	0.09%	1.55%	2.35%	2.92%
Percentage of strain energy shared by crosslinks	19.3%	11.9%	8.7%	6.9%

## 4. Discussion

**Figure 5:**
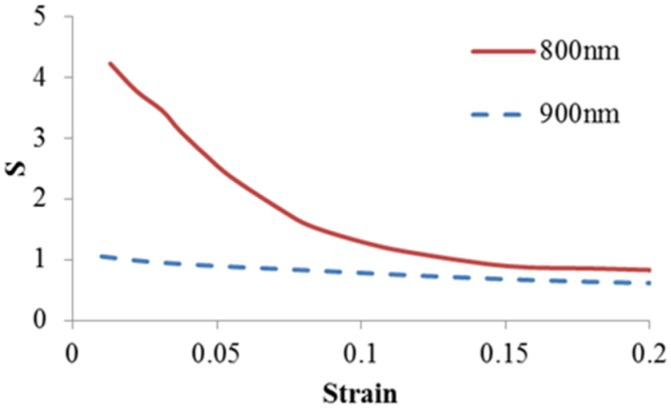
The impact of crosslink threshold on non-affine deformation.

Type I collagen was cross-linked under different conditions to formulate collagen gels for various tissue engineering applications [22]. Cross-linking plays an important role on the mechanical stability of gel. The structural properties of the gel will affect the motility of cells and the function of the regenerated tissue. In this study, a three-dimensional collagen fiber network equivalent to a concentration of 1 mg/mL was developed in a microscale RVE to investigate the role of crosslinks on mechanical responses of collagen gels. The model was validated against published experimental data. The obtained classical strain stiffening effect was elucidated by the role of fiber networking without considering the nonlinear elasticity of collagen fibers. By monitoring the change of fiber orientation angle, the strain stiffening effect could be visualized by continuous fiber alignments. This result is consistent with the experimental study by Vader *et al*. [2] who attributed the strain stiffening of collagen gels to the fiber alignment and densification. It also supports the theoretical hypothesis by Onck *et al*. [16] that strain stiffening in polymer gel was governed by the fiber rearrangement. We also observed the profound reduction of non-affine property S at lower strain, which corresponded to the reconfiguration of random distributed fibers, especially for lower crosslink density or threshold induced relative weak network (Figure 5). The non-affine deformation reduced with stretches corresponding to the gel stiffening. This agrees with the experimental observations by Wen *et al*. [21]. The results, taken together, suggest that the fiber network dominated the strain stiffening of collagen gel. 

The degree of cross-linking could be regulated by parameters including temperature, pH, collagen concentration and adopted polymerization agents [9,10]. In the current study, crosslink threshold, (*i.e.*, the inter-fiber nodal spacing), was used in our model to control the crosslink density. The adopted crosslink threshold from 800 to 1600 nm is based on the study by Lindstrom *et al*. [19]. Our results have demonstrated that the gel stiffness increased approximately 40 times by doubling the crosslink density with the same collagen concentration. A positive bilinear correlation was found between the crosslink density and gel stiffness. The turning point at the crosslink threshold of 1200 nm, *i.e.*, crosslink density of 3.44, was when all fibers were cross-linked. Further cross-linking treatment after this density point would dramatically increase the gel stiffness. This could be explained by the growth ratio of crosslinks/cross-linked fibers within the network. With a crosslink density larger than 3.44, the cross-linked fibers increased 2.8% and the number of crosslinks increased 32.5%, which resulted in a growth ratio of 11.6, compared to the ratio of 3.6 for the crosslink density less than 3.44 (Table 1). This indicated that crosslinks was mainly used to recruit more fibers into the network before the turning point, and then contributed to the reinforcement of fiber network with more crosslinks per node resulting in the pronounced increase in gel stiffness. Even a linear correlation between the crosslink density and engineered tissue stiffness was well accepted [23]. Sheu *et al*. observed a dramatically increase of collagen gel stiffness after fully cross-linking [9]. Gardel *et al*. also demonstrated a nonlinear correlation between the crosslink density and the stiffness of actin fiber network [24]. It should be noted that their crosslink density was calculated as the crosslink mass, which included all the fibers regardless of their crosslinking status. In our work, the crosslink density was based on the amount of cross-linked fibers. However, our adopted definition of crosslink density won’t change the observed bilinear correlation between crosslink density and gel stiffness. Moreover, no experimental evidence has ever demonstrated this bilinear correlation. Our observations might shed lights on future testing and experiments. 

The role of crosslink stiffness on type I collagen gel behaviors was also examined. Corresponding to different chemical structures of crosslinks [10], four crosslink stiffnesses with an increment of 25 MPa (e.g., 25 MPa, 50 MPa, 75 MPa, and 100 MPa), were studied. The gel stiffness increased 28% as the crosslink stiffness increased fourfold (Table 2). This observation aligned with our intuition, and we speculated that the increased gel stiffness was attributed to the increased load sharing capacity of crosslinks. It was calculated that percentage of load shared by crosslinks with stiffness of 25 MPa was 0.09%, compared to 2.92% for crosslink stiffness of 100 MPa. In addition, the percentage of strain energy shared by crosslinks was reduced from 19.3% to 6.9%, which indicated that stiffer crosslinks resulted in much less deformations, which mainly served as the load transmitter. This agrees with the observations by Gardel *et al*. [25] that softer F-actin filaments rather than the stiff crosslinks determined the mechanical response of the network. In addition, our baseline model could be used to illustrate the mechanical behaviors of interfibrillar entanglements for pepsin- and acid-solubilized collagen since crosslinks and entanglements could be considered the same in terms of structure configurations [7]. Compared to the impact of crosslink density, the effect of crosslink stiffness on gel stiffness was insignificant.

The non-fibrous matrix and the statistical estimates of gel stiffness was not considered in our models due to its minimal contribution to the gel mechanics [26]. The fiber curvature and its nonlinearity were also overlooked. In the future, we might consider the potential failure mode of crosslinks at large strains. Regardless of these simplifications, our study has demonstrated the importance of crosslink properties on the mechanical response of collagen gels. Specifically, the delicate microstructural changes in crosslink density and stiffness led to profound change in gel properties. 

## 5. Conclusions

In this study, a RVE model of collagen fiber network was proposed to predict its tensile behavior under various crosslink density and crosslink stiffness. The required model input, such as the dimension, stiffness, or volume fractions of fibers, were adopted from published data. Our model prediction was validated by mimicking a tensile test by Roeder *et al*. [6]. Utilizing the inter-fiber spacing to regulate the crosslink density, we are able to provide some novel insights into the role of crosslink density and stiffness on the mechanical response of type I collagen gel, which can be summarized as:

- The strain stiffening effect of the collagen gel was dominated by the fiber alignment.
- The increased crosslink density has much more impact on the gel stiffening than the crosslink stiffness. A positive bilinear correlation between the crosslink density and gel stiffness was predicted.

These results could improve the understanding of the mechanics of collagen networks for designing and regulating clinical relevant biomaterials. This work could also be extended to study how cells respond to different micromechanical environments.
